# P01.06. The effect of bee venom pharmacopuncture therapy in a neuropathic pain rat model

**DOI:** 10.1186/1472-6882-12-S1-P6

**Published:** 2012-06-12

**Authors:** Y Kwon, M Park

**Affiliations:** 1 Wonkwang University Hospital, Gwangju City, Republic of Korea; 2 Doctor, Gwangju City, Republic of Korea

## Purpose

The purpose was to examine the effect of bee venom (BV) pharmacopuncture therapy with different concentrations on neuropathic pain in a rat model.

## Methods

We performed BV pharmacopuncture therapy with different concentrations on neuropathic pain in a rat model on the 7th day after ligating the L5 nerve as suggested by Kim and Chung. Rats were divided into a control group (treated with normal saline), experimental group I (treated with constantly increased BV concentration, from 1.67×10^-3^mg/kg to 8.35×10^-3^mg/kg, total 3.22×10^-2^mg), and experimental group II (treated fixed high concentration, 3.58×10^-3^mg/kg, total 3.22×10^-2^mg). BV pharmacopuncture was injected to Huantiao (GB30) every other day for 18 days. To identify any therapeutic effect, foot withdrawal threshold to mechanical and thermal stimulation, nerve conduction velocity (NCV), and c-Fos immunological reactivity in the dorsal horn of the spinal cord were analyzed.

## Results

In the pain threshold and the c-Fos immunological reactivity test, experimental group II showed a better therapeutic effect than in experimental group I. In NCV testing, experimental group I showed a better therapeutic effect than experimental group II in the early stage of BV treatment. In the latter stage of BV treatment, however, therapeutic effect was similar in both groups.

## Conclusion

BV pharmacopuncture therapy was effective in neuropathic pain. Under the same total amount of BV dose, treatments with constantly increasing BV concentration and fixed high concentration BV had similar effect.

